# The Relationship Between Language and Social Competence in 3- to 5-Year-Old Children at Risk of and Without Developmental Language Disorder

**DOI:** 10.3390/bs15111536

**Published:** 2025-11-11

**Authors:** Marylène Dionne, Stefano Rezzonico

**Affiliations:** 1 École d’Orthophonie et d’Audiologie, Université de Montréal, Montréal, QC H2E 2G2, Canada; stefano.rezzonico@umontreal.ca; 2 Institut Universitaire sur la Réadaptation en Déficience Physique de Montréal, CIUSSS Centre-Sud-de-l’Ile-de-Montréal, Montréal, QC H2H 2N8, Canada; 3 Centre de Recherche Interdisciplinaire en Réadaptation du Montréal Métropolitain, Montréal, QC H2H 2N8, Canada

**Keywords:** language, pragmatic skills, child, social competence, developmental language disorder, daycare

## Abstract

Developmental language disorder (DLD) is associated with persistent language difficulties that may impact social competence. The aim of this study is to describe the relationship between language, pragmatics, and social competence in French-speaking preschoolers and to identify the specific social competence difficulties observed in children at risk of DLD at this age. The sample included 63 children aged between 36 and 59 months, 12 of whom were at risk of having DLD. Children were assessed using measures of vocabulary, morphosyntax, pragmatic skills, and narrative abilities, while childcare educators completed a questionnaire evaluating social competence. Results revealed that children at risk for DLD exhibited more characteristics related to dependence on adults compared to their peers without DLD. No significant group differences were observed for the other components of social competence. The findings also identified a relationship between pragmatic and personal narrative skills, and social adjustment. These findings support the social adaptation model, suggesting that functional social impacts in children with DLD may arise from limited language abilities rather than an intrinsic socio-emotional disorder. This study highlights the importance of early pragmatic and narrative development in supporting social competence from the preschool age.

## 1. Introduction

### 1.1. Developmental Language Disorder

Communication is at the heart of human experience. Good language skills support one’s social participation. Participation is defined by the International Classification of Functioning, Disability and Health (ICF) as “involvement in a life situation” (World Health Organization, 2001). This framework is used to understand functioning and disability, considering health condition (e.g., a disorder or disease), personal factors (e.g., age, gender), and environmental factors (physical, social, and attitudinal). One of the disorders affecting language and associated with functional impacts in everyday life is developmental language disorder (DLD), a neurological condition of unknown cause found in approximately 7% of children (Norbury et al., 2016). DLD manifests itself in difficulties with speaking, listening, reading, and writing. These difficulties appear at an early age and can be present in one or more language components: phonology, semantics, morphosyntax, and pragmatics (Bishop et al., 2017). The clinical profile may vary depending on the person and their development, but DLD persists throughout life (Bishop et al., 2017). Thus, DLD is characterized by language difficulties that have functional impacts limiting social participation in several areas, including social interactions. People with DLD are at greater risk of experiencing social difficulties, from early childhood through to adulthood, than their peers without DLD. Social competence is closely linked to language (Junge et al., 2020; Wieczorek et al., 2024). To interact, one must understand the messages being conveyed, state a message to convey one’s ideas, and have good conversational skills.

### 1.2. Social Competence in DLD

Social competence is defined as effectiveness or skill in social interactions (Hwa-Froelich, 2014; Rose-Krasnor, 1997). It includes the necessary skills, such as social cognition, emotional skills, self-regulation, and communication (Hwa-Froelich, 2014; Junge et al., 2020), that support a person’s social adjustment (fulfilling social roles and personal satisfaction) and their social functioning (adaptation and behavior) in their interactions (Ashton, 2018; Breault, 2023). Social competence difficulties in terms of social adjustment are often described as internalized difficulties, while those in terms of social functioning are usually referred to as externalized difficulties. People with DLD tend to have more internalized difficulties such as higher anxiety levels and lower self-esteem, which can lead to depressive symptoms (Beitchman et al., 2001; Conti-Ramsden & Botting, 2004; Fujiki et al., 1999). Children with DLD tend to be more passive and dependent on adults to manage their friendships and plan outings (Conti-Ramsden & Durkin, 2008; Liiva & Cleave, 2005; P. C. McCabe, 2005). They are at greater risk of social isolation, since they form fewer friendships, have fewer positive social interactions, and are at greater risk of being bullied (Conti-Ramsden & Botting, 2004; Craig, 1993; Dubois et al., 2020; Durkin & Conti-Ramsden, 2007; Fujiki & Brinton, 2014; Fujiki et al., 1996). DLD is also associated with more externalized difficulties, but the profile is less clear in the literature (Chow, 2018; Donolato et al., 2022; Yew & O’Kearney, 2013). Some studies found higher level of aggressive behaviors in the social interactions of young children with DLD (Bakopoulou & Dockrell, 2016; Brinton et al., 2000; Maggio et al., 2014; P. C. McCabe, 2005; Özcebe et al., 2020; Willinger et al., 2003) while other studies did not find a difference (Brinton et al., 2000; Joffe & Black, 2012; P. C. McCabe & Meller, 2004; Redmond & Rice, 1998; Stanton-Chapman et al., 2007). Children with DLD sometimes manage conflicts less effectively and use strategies that are considered more resistant (Brinton & Fujiki, 2014; Craig, 1993; Fujiki & Brinton, 2014; P. C. McCabe, 2005) despite having prosocial behaviors (Dubois et al., 2020; Fujiki & Brinton, 2014; Toseeb et al., 2017). The only externalized difficulties that seem to be more widely agreed upon are attention difficulties and hyperactivity, which is not surprising given the high co-occurrence of DLD and ADHD (Snow & Douglas, 2017; Timler & White, 2014; Yew & O’Kearney, 2013).

Social competence may vary depending on the context (where and with whom) in which interactions occur (Junge et al., 2020; Rose-Krasnor & Denham, 2008). For example, a child’s social adjustment may be perceived as satisfactory by their parents at home, while the same child may be perceived as having more internalized difficulties by their educator or teacher. One particularly significant context for social interaction for many preschoolers is daycare, as it offers frequent opportunities to engage with their peers and adults. Children with DLD are therefore at increased risk of experiencing functional social impacts in this setting, such as during circle time, daily routines, and play activities.

### 1.3. Developmental Trajectory and Explanatory Models

In summary, the literature suggests that children with DLD have a prosocial profile but may have more emotional, social, and behavioral difficulties and be less independent in their social interactions. More recently, longitudinal studies following the social competence of children with DLD showed that language abilities at a younger age play a predictive role in their later social adjustment and functioning (Conti-Ramsden & Botting, 2004; Conti-Ramsden et al., 2019; St Clair et al., 2011). The few existing longitudinal studies also showed a heterogeneous developmental trajectory. For some children with DLD, social competence difficulties appear at an early age. For others, these difficulties become more apparent during adolescence, while for others, the functional impacts in this area are much less significant throughout the developmental period (Beitchman et al., 2001; Brinton & Fujiki, 2014; Conti-Ramsden et al., 2019; Mok et al., 2014). However, most of these studies were conducted during school age or adolescence, whereas clinically, functional impacts are observed as early as the preschool period (P. C. McCabe, 2005). Studies on developmental trajectories tend to corroborate developmental models that claim that socio-emotional disorders observed in older children and adolescents with DLD are not intrinsic, as the social adaptation model originally proposed by Redmond and Rice (1998) suggests. Models that Redmond and Rice (1998) labeled as social deviance models claim that socio-emotional difficulties are intrinsic to this population, and therefore, these difficulties should be consistent difficulties and be stable over time. The social adaptation model (Redmond & Rice, 1998) states that the socio-emotional and behavioral difficulties reported by the school stemmed from a difficulty in using limited language resources in a more demanding communicative context and not from an intrinsic socio-emotional disorder.

In line with a social adaptation model (Redmond & Rice, 1998), early language intervention for children with DLD could help support the social participation of people with DLD. Thus, it is crucial to identify which specific aspects of language are related to social competence. While links between language and social competence exist, the nature of this relationship remains to be clarified. One of the language components often associated with social competence is pragmatics: a set of skills that enable people to understand and use language in context, including conversational and discursive skills, communicative intents, inferences, and turn-taking (Airenti, 2017). Receptive and expressive difficulties with structural language (vocabulary/morphosyntax) have a greater impact on academic success, while weaker pragmatic skills are more closely associated with difficulties in social interactions and behavioral and emotional difficulties (Benner et al., 2002; Conti-Ramsden et al., 2019; Liiva & Cleave, 2005; Mok et al., 2014; Reed, 2017; Snow & Douglas, 2017; St Clair et al., 2011). However, few studies have explored the relationship between language and social competence at the preschool age. Given the heterogenous and evolving nature of language difficulties, this relationship may not be the same throughout development.

In summary, the social competence profile of preschoolers with DLD remains understudied. The lack of data at this age makes it difficult to understand when social competence difficulties start to occur and whether the same language aspects are associated with social competence.

### 1.4. Aim of This Study and Hypothesis

The aim of this study is to describe the relationship between language, pragmatics, and social competence in francophone preschool children (aged 3 to 5) at risk of and without DLD. Two research questions are of particular interest to us: (1) What is the social competence profile of preschoolers at risk of DLD at daycare? (2) What language components are associated with social competence in preschool children?

We measured children’s lexical diversity, morphosyntax, pragmatics, personal narrative, and social competence. We first hypothesized that children at risk of DLD would report significantly lower language and pragmatic skills than their typical language-developing peers. According to Redmond and Rice’s (1998) social adaptation model, we hypothesized that the children at risk of DLD in our study will have lower social competence skills but not more marked than their peers, unlike older children with DLD. Finally, we hypothesize that children with better pragmatic skills will demonstrate better social competence. This is in line with previous research of school-age children and adolescents that indicated that pragmatic skills are linked to the development of social competence (Conti-Ramsden et al., 2019; Junge et al., 2020; P. C. McCabe, 2005).

## 2. Materials and Methods

A cross-sectional experimental model was chosen to meet the aim of this study. Daycare centers in the Tiohtiá:ke (Montréal, Québec, Canada) area were contacted by telephone or by email by the first author to take part in this study. The director was informed (verbally or by an information letter) that we were recruiting groups of children in daycare centers with at least one child at risk of DLD. When this was the case, an information letter addressed to the parents of the children in the group was sent to the director, who then contacted the parents. Parents who agreed to their child taking part in the project could then contact our team to fill in the consent form. In total, 5 daycares centers were recruited.

The data presented in this study stems from a larger intervention study that started in November 2019. In the context of the COVID-19 pandemic, changes had to be made to post-pandemic data collection procedures (described below).

### 2.1. Participants

In total, 66 children were recruited. To be included in this study, children had to be between 36 and 71 months old, attend a French-speaking daycare center, and have language development deemed typical or be at risk of DLD. Children with a biomedical condition likely to be associated with a language impairment (e.g., autism, deafness) were excluded. Monolingual or bilingual French-speaking children were eligible to participate. To be considered bilingual, the child had to be able to interact (speak and understand) in French and at least in one other language. Three children were excluded from this study because they did not meet the inclusion criteria (one child was learning French and was unable to interact in French, one child was undergoing an autism assessment, and another child had moderate hearing loss). One child was 34 months old but was in a group with children aged 36 to 42 months in their daycare. Since all children from this group participated in the larger intervention project, this child was included in this study.

Parents completed a socio-demographic questionnaire and indicated whether their child had language difficulties confirmed by an assessment conducted by a registered speech-language pathologist. This information was used to form the following two groups: *typical language* and *at risk of DLD*^1^. The results from this study’s language tasks were also used to ensure that language difficulties did not go unnoticed in children whose development was judged by their parents to be typical. The final sample is therefore composed of 63 children aged from 34 to 63 months (*M* = 49.43, *SD* = 7.35), including 12 at risk of having DLD. From this sample, 28 were assigned female at birth, 35 were assigned male at birth, 40 were monolingual French-speaking, and 23 were bilingual. No statistical differences were found between both groups (typical language and at risk for DLD) for age, gender, linguistic status, and socio-economic status. Participants’ characteristics are presented in Table 1.

### 2.2. Measures

#### 2.2.1. Language: Pragmatics and Personal Narrative

Parents completed the French version of the Language Use Inventory questionnaire (LUI-French; Pesco & O’Neill, 2018). This questionnaire provides a global assessment of pragmatic skills in children aged 18-47 months. It measures why and how children communicate and what they communicate about. The LUI-French comprises 177 items distributed across 14 subscales (Pesco & O’Neill, 2018). The LUI-French Total Score is calculated from the results obtained on 10 subscales (maximum raw score = 159): C: Types of words your child uses; D: Your child’s requests for help; F: How your child uses words to get you to notice something; G: Your child’s questions and comments about things; H: Your child’s questions and comments about people; I: You child’s use of words in activities with others; J: Teasing and your child’s sense of humour; K: Your child’s interest in words and language; M: How your child’s adapts conversation to other people and N: How your child is building longer sentences and stories. There are currently no standardized normed data for the LUI-French. Nevertheless, like the original English version, it has demonstrated excellent psychometric qualities, in particular, a high level of sensitivity and specificity, making it possible to distinguish between children whose development of pragmatic skills is judged to be typical or not (Pesco & O’Neill, 2016; Pesco & O’Neill, 2023).

Spoken communicative intents, a specific pragmatic skill, were assessed using a semi-structured symbolic play task, the Neighbourhood Game (Sylvestre et al., 2023). In this task, the child plays with the experimenter according to a predetermined scenario in which 8 communicative intents are solicited on at least four occasions: suggesting, protesting, reporting an event, sharing preferences, accompanying actions, announcing actions, explaining, and arguing. A communicative intent is considered acquired if expressed at least three times by the child, emergent if expressed once or twice, and absent from the repertoire if never expressed (maximum raw score = 8). There is no standardized normed data for this task.

The child’s discourse skills were measured using a personal narrative (PN) task based on a procedure adapted from A. McCabe and Rollins (1994). The experimenter first told the child a PN on various themes (helping an injured child, a broken toy, and making a mess during an art project) to engage the child and provide a model. Then, they encouraged the child to express their own PN (Dionne et al., submitted). When the child indicated the end of their story, the experimenter asked questions, if necessary, to clarify certain parts of the story grammar: what happens, who is involved, where and when the story takes place, the high point, the solution or the elements following the high point, and the final state (A. McCabe et al., 2008; Peterson & McCabe, 1994). A score reflecting the macrostructure level of the story produced with the help of the experimenter was then provided to the child’s PN based on a high-point analysis inspired by a procedure from A. McCabe et al. (2008). The maximum score of 7 represents a complete and classic PN, a score of 6 indicates a PN that ends at the high point, a score of 5 indicates a chronological PN who does not include the high point, a score of 4 indicates a leapfrog PN, a score of 3 indicates a miscellaneous PN, a score of 2 indicates a PN that contains 2 unrelated events, a score of 1 indicates a PN that contains only one past event, and a score of 0 indicates a PN that contains no past event or no production.

#### 2.2.2. Language: Lexical Diversity and Morphosyntax

The VOCD-D measure was used as a measure of the child’s lexical diversity. VOCD-D is a computerized measure that represents the probability that a new word will be introduced into a corpus of increasing length (MacWhinney, 2000). A high VOCD-D score indicates greater lexical diversity. In this study, the VOCD-D was calculated from the transcripts of the PN task (Dionne et al., submitted). Morphosyntax was assessed using the mean length of utterances calculated in morphemes (MLUM). The MLUM is generally a more global measure of morphosyntactic skills but is considered a fair clinical marker for DLD (Thordardottir et al., 2011). The MLUM was also calculated from the transcripts of the PN task (Dionne et al., submitted).

#### 2.2.3. Social Competence

Social competence was measured using the original French version of the Social Competence and Behavior Evaluation Scale (SCBE; Dumas et al., 1997), a questionnaire completed by the child’s educator to assess social competence and adaptation difficulties in children aged 30 to 78 months. The SCBE (Dumas et al., 1997) is composed of 80 items, rated on a 0 to 5 Likert scale, which assess how children express their emotions and self-regulate, how they interact with their peers, and how they interact with their childcare educator. The 80 items are then used to calculate a score for 8 basic scales: Depressive–Joyful, Anxious–Secure, Isolated–Integrated, Dependent–Autonomous, Angry–Tolerant, Aggressive–Calm, Egotistical–Prosocial, and Oppositional–Cooperative (maximum score for each scale = 50). It is also possible to calculate a score for four global scales: General Adaptation, Social Competence, Internalizing Problems, and Externalizing Problems. The Internalizing Problems global scale comprises 20 items on anxiety, depression, social isolation, and dependence on the educator, thus representing the child’s social adjustment difficulties. The Externalizing Problems global scale comprises 20 items on irritability, aggressivity, selfishness, and opposition to others, thus representing the child’s social functioning difficulties. A high score on a basic or global scale places the child on the more positive pole, while a lower score indicates difficulties.

### 2.3. Procedure

Data were collected in the child’s daycare by the first author, who is a registered speech therapist in Quebec. For the 21 children who took part in this study pre-pandemic, three PNs were elicited (theme of the PN model was in a random order). For the 42 children who took part in this study post-pandemic, one PN was elicited (theme of the model was “a mess during an art project”). Then, all children participated in the Neighbourhood Game (Sylvestre et al., 2023) with the experimenter. Both tasks were video recorded to allow transcription and subsequent analysis. At the same time, the LUI-French (Pesco & O’Neill, 2018) was given to the parents, and the SCBE (Dumas et al., 1997) was given to the childcare educators. They were permitted one week to complete the questionnaires.

### 2.4. Data Analysis

The LUI-French (Pesco & O’Neill, 2018), the Neighbourhood Game (Sylvestre et al., 2023), and the SCBE (Dumas et al., 1997) were scored as per the manual. All PNs were transcribed by the first author and two research assistants using the Computerized Language Analysis software (CLAN, version 23-May-2023, Pittsburgh, United-States, MacWhinney, 2000) and following the CHAT conventions (MacWhinney, 2000; Parisse & Morgenstern, 2010, for the French transcription). PN high-point scores were calculated by the first author and a research assistant. Inter-rater agreement was carried out on all the data. Where there were major disagreements, a third expert was brought in to reach a consensus. The means of VOCD-D, MLUM, and PN high point were calculated for the children who produced more than one PN (pre-pandemic data collection).

As only the SCBE is validated with standard scores, raw scores for all measures were converted into Z scores. This also allowed the use of the LUI-French (Pesco & O’Neill, 2018) scores for children who were older than 47 months. To do so, the mean and standard deviation for the group of typical language children who were between 3 and 4 years old and who were between 4 and 5 years old were calculated for all measures. A distinction between the 3- to 4-year-old and 4- to 5-year-old groups was considered relevant, since the SCBE (Dumas et al., 1997) suggests that younger children obtain lower scores. The Z score was calculated by comparing each child’s score with the descriptive data for their age group (Z = [child’s score—mean]/standard deviation).

Data were compiled in an Excel file (version 16.0, Microsoft Corporation, Redmond, WA, USA) and then analyzed using JASP software (version 0.17.2.1, Amsterdam, Netherlands, JASP Team, 2020). Bayesian analyses were carried out. Bayesian statistics do not include a p-value but a Bayesian factor (BF) indicating both the direction and the strength of the evidence (size effect) in favor of H_1_ or H_0_. Jeffreys’s (1961) classification criteria, as reported in Table 2, were used to interpret the value of Bayesian factors in favor of H_1_ (BF_10_) and quantify the strength of evidence (Brydges & Gaeta, 2019; van Doorn et al., 2021). For instance, a BF_10_ of 11 indicates that Groups A and B are different and that there is strong evidence in support of this hypothesis (H_1_). On the other hand, a BF_10_ of 0.06 indicates that Groups A and B are similar and that there is strong evidence in support of this hypothesis (H_0_). BF_10_ values close to 1 indicate lack of evidence to support either H_1_ or H_0_. Imbalanced and small samples might limit the possibility of observing strong evidence for H_1_ or H_0_.

To answer our first research question, a series of Bayesian Mann–Whitney t-tests were performed to compare the means of the two groups (children with typical language and children at risk for DLD) for all measures. A Cauchy distribution centered at 0 with a scaling parameter of 0.707 was used for the a priori distribution, following the recommendation of Rouder et al. (2009) and as the default value in JASP when no specific priors are available (Wagenmakers et al., 2018). The H1 model was compared to the H0 model to determine whether the former provided a better explanation of the a posteriori distribution. The alternative hypothesis was that the group of children with typical language would have higher scores than the group of children at risk for DLD.

To answer our second research question, nonparametric Bayesian correlational analyses (Kendall’s tau-b) were first performed to explore the relationship between social competence and all language measures. The H1 model (assumption of correlation between variables) was compared to the H0 model (absence of correlation). The following criteria were used to quantify the strength of Kendall’s correlations: tau-b ≥ 0.71 = very strong correlation, tau-b ≥ 0.49 = strong correlation, tau-b ≥ 0.26 = moderate correlation, and tau-b ≥ 0.06 = weak correlation (Wicklin, 2023). A beta distribution of width 1 was used for the a priori distribution. Then, to better understand what language components are associated with social competence in preschool children, multiple regression analyses were carried out. Because specific priors were not available, we used the default parameters in JASP (which are based on Rouder et al., 2012). Thus, a Jeffreys–Zellner–Siow (JFZ) a priori distribution with an r-scale value of 0m354 (default setting in JASP) was used. In order to identify the most plausible model given the data, the possible models were compared to a null model, and the model with the highest BF_10_ was retained. Supplemental considerations were added using posterior summaries inspection. Inclusion BF indicated how much evidence for a factor there was across all models, and credible intervals were inspected. Given the exploratory nature of the research, a 90% CI was set for inspection. Nonparametric tests were used because several variables did not meet the assumptions associated with parametric tests (equality of variances, normality, or homoscedasticity).

## 3. Results

### 3.1. Differences Between Typical Language and at Risk for DLD Groups

Table 3 presents the results obtained by children at risk for and without DLD for each of the language measures. Table 4 presents the results obtained by children for both groups for the social competence scales. Raw scores are presented in both tables. Because the Z scores were calculated from the means of the 3- to 4-year-olds and then the 4- to 5-year-olds, the results are separated this way in each of the tables.

Table 5 presents the BF_10_ values for t-tests comparing the results between the typical language and at risk for DLD groups on all language and social competence measures.

These tests identified differences between the two groups. They indicate a strong probability of differences between the groups for the LUI-French Total Score (*W* = 510.5; BF_10_ = 23.992), as well as for 6 of the 10 subscales: C (*W* = 460.5; BF_10_ = 12.351), H (*W* = 471.5; BF_10_ = 11.013), J (*W* = 480.5; BF_10_ = 14.426), K (*W* = 492.5; BF_10_ = 15.846), M (*W* = 501.5; BF_10_ = 32.186) and N (*W* = 502.5; BF_10_ = 37.675). Strong probability of group difference was also observed in the production of the PN (*W* = 517.5; BF_10_ = 11.576), whereas moderate probability of group differences was observed in the production of spoken communicative intents in the Neighbourhood Game (*W* = 470; BF_10_ = 6.232) and, for the lexical measure, in the VOCD-D (*W* = 445.5; BF_10_ = 3.695). These results highlight the lexical and pragmatic difficulties of children at risk of DLD, since this group had lower scores than the comparison group on all language measures. Anecdotal evidence in favor of H_1_ was found for MLUM (*W* = 469; BF_10_ = 2.837), suggesting that our sample may not have been sufficient to clearly demonstrate whether the groups were different or not.

Regarding social competence, there was a group difference in only 2 of the 12 scales in the Social Competence and Behavior Evaluation (SCBE) questionnaire (Dumas et al., 1997): the Internalized Problems scale (*W* = 463; BF_10_ = 4.019) and the Dependent–Autonomous scale (*W* = 477; BF_10_ = 10.738). Children at risk for DLD had lower scores on both scales, suggesting that these children have more internalized problems and a more dependent profile than their peers without language difficulties. On the other hand, the results highlight the relative strengths of the at risk for DLD children in our sample in terms of social competence, since there is moderate evidence in favor of H_0_ (no group difference) for four scales of the French SCBE (Dumas et al., 1997): the Externalizing Problems (*W* = 255.5; BF_10_ = 0.192), Aggressive–Controlled (*W* = 233.5; BF_10_ = 0.234), Selfish–Prosocial (*W* = 253.5; BF_10_ = 0.169), and Resistant–Cooperative (*W* = 294; BF_10_ = 0.288) scales. In fact, the group of children at risk for DLD scored similarly, and sometimes even higher, than the group of children without language difficulties on these four measures. Regarding the other subscales, our sample was not sufficient to position strongly in favor of H1 or H0. Thus, no conclusion can be drawn about the trend towards a group difference on the Anxious–Secure (*W* = 422; BF_10_ = 1.531) and Isolated–Integrated (*W* = 422.5; BF_10_ = 1.507) subscales nor on the similarities between groups on the Depressive–Joyful (*W* = 406.5; BF_10_ = 0.920) and Angry–Tolerant (*W* = 330; BF_10_ = 0.492) subscales.

### 3.2. Associations Between Language and Social Competence

Table 6 presents the correlation results between the SCBE scales and language measures for which the strength of evidence is at least moderate (BF_10_ ≥ 3, n = 63).

The results of the correlational analyses were used to select the variables included in the multiple linear regression analyses carried out to determine which language measures were most associated with the social competence of the children in the sample. First, an analysis was carried out with the SCBE global Internalized Problems scale (Dumas et al., 1997) as the dependent variable, since a difference between both groups was found for this scale. Both linguistic measures (MLUM and VOCD-D) were included. For pragmatic measures, the J, K, and M subscales of the LUI-French (Pesco & O’Neill, 2018), as well as the high-point measure from the PN task, were chosen given that a correlation was present with the Internalized Problems scale. The N subscale of the LUI-French (Pesco & O’Neill, 2018) was also added to the regression model, since both correlations with the global Internalized Problems scale (τ*b* = 0.216, BF_10_ = 2.839) and the Isolated–Integrated basic scale (τ*b* = 0.213, BF_10_ = 2.605) were close to the moderate evidence threshold in favor of H_1_ (BF_10_ ≥ 3). The production of spoken communicative intents was also added, as it allows us to see the potential effects of pragmatic skills as measured in an ecological task (symbolic play). The results indicate that a linear combination of three variables, namely the scores on the M and N subscales of the LUI-French (Pesco & O’Neill, 2018), as well as the score for the high-point analysis in the PN task (Dionne et al., submitted), represents the most plausible model to explain the score on the Internalized Problems scale (Dumas et al., 1997, BF_10_ = 2670.696). The strength of evidence for this model is very strong. This model explains 39.4% of the observed variance. The three variables were the only ones to show *Inclusion BF* higher than 3, indicating consistent evidence for these three variables across models. The three variables’ CI set at 90% also excluded 0.

Second, an analysis was carried out with the SCBE global Externalized Problems scale (Dumas et al., 1997) as the dependent variable. Both linguistic measures (MLUM and VOCD-D) were included, along with the I and M subscales of the LUI-French (Pesco & O’Neill, 2018). The I subscale was chosen because there was a correlation with the global Externalized Problems scale, whereas there was a correlation with the Oppositional–Cooperative basic scale. The results indicate that none of the models better explained the outcome on the global Externalized Problems scale compared to the null model.

## 4. Discussion

The aim of this study was to describe the relationship between language, pragmatics, and social competence in francophone preschool children (aged 3 to 5) with and without DLD. This relationship remains little explored at preschool age and even less so in terms of how it is actualized in an important living environment—their daycare—for the young child. First, our results identify the pragmatic and social competence profile of preschoolers at risk of DLD. Then, it describes what language components are associated with social competence at this age.

### 4.1. What Is the Pragmatic and Social Competence Profile at Daycare of Preschoolers at Risk of DLD?

Our results highlight the global pragmatic difficulties of children at risk of having DLD, since they score significantly lower on the three global scales of the LUI-French parent questionnaire (Pesco & O’Neill, 2018), as well as on 6 of the 10 subscales: C: Type of words; H: Questions/comments on self/others; J: Talk in activities with others; K: Interest in words and language; M: Adapting conversation to others; and N: Building longer sentences and stories. These results highlight that a significant gap develops between the pragmatic skills of children at risk for DLD and their neurotypical peers from preschool age. Differences between groups, and the strength of evidence, are higher for the last two subscales, which focus on children’s conversations (M) and words used in complex sentences and stories (N). Narrative difficulties of children at risk for DLD are also present in the results of the PN task (Dionne et al., submitted), since this group produces less complete narratives than their peers without language difficulties. Previous studies have reported macrostructure difficulties in fictional narratives of school-aged children with DLD (Gillam et al., 2018). This study identifies difficulties in the production of another type of narrative: personal narrative. It also reveals that these difficulties can be present as early as preschool age. Children at risk for DLD also scored lower on the Neighbourhood Game task, suggesting that they produce less spoken communicative intent than their peers without language difficulties. This indicates that they encounter pragmatic difficulties in at least two of their living contexts: at home, as perceived by parents, and in their daycare environment, via an ecological task. In summary, children at risk for DLD present pragmatic difficulties that are significant from preschool onwards and which would benefit from support through early intervention. Moreover, the difficulties tend to be generalized to conversation, narrative, and communicative intent, and are not specific to any pragmatic skill.

Regarding social competence, interestingly, there is no difference between the groups in terms of the child’s general adaptation (global score). It therefore appears that in early childhood, social competence difficulties are not generalized. Rather, they seem to affect internalizing difficulties, more specifically, autonomy, since children at risk for DLD are, as a group, more dependent on their childcare educator in their social interactions. These findings are consistent with previous studies that have also identified autonomy difficulties in preschoolers at risk for DLD (Bakopoulou & Dockrell, 2016; P. C. McCabe, 2005). Children at risk for DLD from our sample, however, do not present more emotional difficulties than their peers nor are they perceived as being more isolated. The emotional well-being profile and the level of social isolation in preschoolers at risk for DLD is currently unclear in the literature. Our findings support studies that have not found greater social and emotional difficulties for these children at this age (Gregl et al., 2014; Levickis et al., 2018) and they align with the social adaptation model (Redmond & Rice, 1998). This supports the idea that social competence difficulties are not an intrinsic disorder in children with DLD and may appear more pronounced during school age or adolescence (Conti-Ramsden et al., 2019; St Clair et al., 2011) as language demands become increasingly challenging. It is still important to note that post hoc analyses showed greater variability within the at risk for DLD group. While some children demonstrated strong socio-emotional adjustment, a notable proportion exhibited marked difficulties. This increased inter-individual variability may indicate underlying vulnerabilities in social–emotional development that are present in early childhood for some children at risk for DLD and that are not yet salient compared with their peers without language difficulties.

It is also interesting to note that, in this study, children at risk for DLD showed similar social functioning to their peers without language difficulties, since they do not show more externalized difficulties. There is no consensus in the literature on the profile and trajectory of social functioning in preschoolers at risk for DLD. In this study, the strength of the evidence allows us to state with greater certainty that no real difference is present between both groups, corroborating results from previous studies that have identified little or no behavioral difficulties at preschool age (Brinton et al., 2000; Redmond & Rice, 1998; Stanton-Chapman et al., 2007). In fact, post hoc analyses revealed that all children from the at risk for DLD group scored within the typical range on the Externalizing Problems global scale of the SCBE (Dumas et al., 1997). However, the present study did not measure social functioning difficulties related to attention and hyperactivity. It would have been interesting to assess whether the social competence of children at risk for DLD is affected by attentional difficulties, especially as these are the behavioral manifestations most frequently associated with these children (Snow & Douglas, 2017; Timler & White, 2014; Yew & O’Kearney, 2013).

### 4.2. What Language Components Are Associated with Social Competence in Preschool Children?

The correlational analyses show that several pragmatic and discursive skills are associated with children’s social competence skills as measured by the SCBE (Dumas et al., 1997). In fact, five characteristics of social competence are associated with at least one pragmatic or narrative skill: the basic scales of Depressive–Joyful, Anxious–Secure, Isolated–Integrated, Dependent–Autonomous, and Oppositional–Cooperative. As for the linguistic measures (MLUM and VOCD-D), they are only associated with the Dependent–Autonomous basic scale. These results are in line with our original hypothesis that children with better pragmatic skills would have better social competence. They are also in line with previous studies that have identified links between pragmatics and social competence in older children, highlighting the importance of early pragmatic development (Conti-Ramsden et al., 2019; Junge et al., 2020; St Clair et al., 2011). In previous studies, however, the specific pragmatic skills involved in social competence are rarely specified (van der Wilt et al., 2019). Our study helps filling this gap, as the multiple linear regression analysis revealed that it is conversational skills, the use of longer sentences and stories, and the production of the high point in PN that best explain the social adjustment profile (internalized problems) that distinguishes children at risk for DLD and those without language difficulties. Conversation and storytelling are frequent activities in young children’s social interactions. They are also complex tasks that rely not only on linguistic but also on cognitive, pragmatic, and sociocultural abilities (Verhoeven, 2010). Many children with DLD depend on the support from an adult to accomplish their social roles (de Weck & Rosat, 2003; Paul et al., 2018; Troia et al., 2023). Our results allow us to hypothesize that conversational and narrative difficulties lead children to be more isolated, less autonomous, and to experience fewer satisfying interactions, which has an impact on their social adjustment. According to Snow and Douglas (2017), the pragmatic language competences are dependent on linguistic abilities (phonology, morphology, syntax, and vocabulary), executive functions (i.e., planning, self-monitoring, etc.) and social cognition functions (i.e., theory of mind, emotion recognition, inferencing, etc.), and the psychosocial impacts are also dependent on psychological characteristics and the social and environmental context. We could then speculate that mild deficits in executive functions and social cognition functions in children with DLD (i.e., Niu et al., 2024; Schwartz Offek & Segal, 2022), along with linguistic difficulties, contribute to an impact on the pragmatic competence of children at risk of DLD. Their pragmatic abilities, in turn, might impact on meaningful social interaction that foster further linguistic and socio-emotional development leading to the socio-emotional profiles described in adolescents (Conti-Ramsden et al., 2019). The results of the present study do not allow us to comment further on the specific role of those components on pragmatics skills of children with DLD. From a speech and language pathology point of view, this better understanding of the role of conversational and narrative skills from preschool age is important to consider when putting in place early interventions likely to prevent the onset of internalized difficulties.

The fact that the multiple linear regression analysis did not identify a model that better explains the variance observed in social functioning (Externalizing Problems) compared to the null model is contrary to our initial hypothesis. The few previous studies that explored the link between language and, specifically, externalizing difficulties in older children have found associations between pragmatics and higher levels of hyperactivity (Leonard et al., 2011; St Clair et al., 2011; van den Bedem et al., 2018). However, hyperactivity difficulties were not measured in our study. Further studies are therefore needed to understand whether pragmatics difficulties play a role in social functioning difficulties from preschool age or whether the relationship is different from the one observed in older children.

### 4.3. Strengths, Limitations, and Future Direction

To our knowledge, this is the first study to describe the pragmatic and social competence profile of French-speaking preschoolers at risk for DLD. It is also the first study to describe the relationship between language and social competence in an educational setting important for young French-speaking children—their daycare. A better understanding of preschoolers’ pragmatic and social difficulties can help childcare educators to better identity early language difficulties and provide support when needed. This study is also among the few to explore the role of personal narrative abilities in French preschoolers. While fictional narrative is known to play an important role in academic success, personal narrative is the most frequent type of discourse among preschoolers and deserves further exploration.

Our study has certain limitations, including the generalization of results to French-speaking children in Quebec. Only 12 children at risk for DLD were included in the sample. The clinical profile of DLD can be heterogeneous at an early age, which may have led to variability in the data collected. It should be noted, however, that despite the small number of participants, the Bayesian approach that was used allowed us to quantify the probability that the results obtained indicate a difference or a lack of evidence to reject H_0_ (Brydges & Gaeta, 2019; van Doorn et al., 2021). For several of our results, the strength of the evidence was moderate to strong for both H1 and H0 despite the small sample size and group imbalance. However, the small sample and the imbalance between groups might have an impact on some of the results that showed anecdotal evidence, namely some subscales of the Internalizing Problems scale and one subscale of the Externalizing Problems scale. For instance, a larger and more balanced sample could reveal differences between the two groups on the Depressive–Joyful, Anxious–Secure, Isolated–Integrated, and Angry–Tolerant subscales. A deeper look into the data showed an important individual variability for these scales which can also contribute to the lack of evidence in support of H_1_. In addition, the results describing the relationship between language and social competence were obtained with the whole sample (children at risk for DLD and without language difficulties). Some limitations are also present regarding the measures used. The linguistic measures used (VOCD-D and MLUM) were dependent on the performance in the PN task (Dionne et al., submitted). To better judge children’s lexical and morphosyntactic levels, standardized tests should have been administered. In fact, this was initially included in our original protocol, but we had to remove certain tasks to reduce the time spent with children due to public health restrictions imposed during the COVID-19 pandemic. Furthermore, in future studies, it would be relevant to add a task assessing children’s verbal comprehension, since previous studies have suggested links between this skill and social competence. Finally, social competence was measured in a single context, namely in daycare, by the childcare educator. However, social competence can manifest itself differently depending on the context (Rose-Krasnor, 1997). Future studies should assess social competence in several settings (e.g., by parents at home and by the childcare educator) to better understand whether the functional impacts experienced by children at risk for DLD, such as being more dependent, manifest themselves in the same way depending on the context.

## 5. Conclusions

This study lays the groundwork for a better understanding of the relationship between language, pragmatics, and social competence in francophone preschool children with and without DLD. It highlights the social competence profile of these children in a significant setting, the daycare, as well as the importance of the understudied role of pragmatics and personal narrative. As predicted by the social adaptation model (Redmond & Rice, 1998), difficulties in the social competence of children with TDL seem to be less salient in early childhood than in school age or adolescence. This invites us to explore early interventions that support pragmatic and narrative skills with the aim of sustaining the child’s autonomy.

## Figures and Tables

**Table 1:** Participant characteristics (*N* = 63).

	Typical Language	At Risk for DLD	Total
**Number of children**	51	12	63
**Sex at birth**			
Female	23	5	28
Male	28	7	35
**Linguistic status**			
Monolinguals	37	3	40
Bilinguals	14	9	23
**Age (in months)**			
Mean (standard deviation)	48.94 (7.33)	51.5 (7.34)	49.43 (7.35)
Range	34–63	39–61	34–63

**Table 2:** Bayes factor in favor of H_1_ values and interpretation according to Jeffreys’s (1961) criteria.

BF_10_ Value ^1^	Interpretation
>100	Very strong evidence in favor of H_1_
10–100	Strong evidence in favor of H_1_
3–10	Moderate evidence in favor of H_1_
1–3	Anecdotical evidence in favor of H_1_
0.333–1	Anecdotical evidence in favor of H_0_
0.1–0.333	Moderate evidence in favor of H_0_
0.01–0.1	Strong evidence in favor of H_0_
<0.01	Very strong evidence in favor of H_0_

^1^ BF_10_ = Bayes factor in favor of alternative hypothesis.

**Table 3:** Mean scores for language measures (raw scores).

Measure	Group	*N*	Mean	S.D.	Min.–Max.
		**Children aged 3 to 4 years**			
*N* total	T.D.	26			
	DLD	4			
MLUM	T.D.	25	5.016	1.507	1–7.585
	DLD	4	1.342	1.064	0–2.5
VOCD	T.D.	25	34.896	10.578	0–51.52
	DLD	4	2.815	5.630	0–11.26
Neighbourhood Game	T.D.	25	4.72	1.86	0–8
	DLD	4	0.25	0.5	0–1
Personal narrative	T.D.	25	4.12	1.697	0–7
	DLD	4	1	2	0–4
Language Use Inventory—French					
Total Score	T.D.	24	142.458	11.24	119–159
	DLD	4	102.25	27.693	64–125
Part 2: Word	T.D.	24	29.375	1.173	25–30
	DLD	4	27.75	3.202	23–30
Part 3: Longer sentences	T.D.	24	113.083	10.762	91–129
	DLD	4	74.5	24.745	41–96
C: Types of words	T.D.	24	22.667	0.761	20–23
	DLD	4	21.5	1.732	19–23
D: Requests for help	T.D.	24	6.708	0.624	5–7
	DLD	4	6.25	1.5	4–7
F: getting someone to notice something	T.D.	24	5.5	0.659	4–6
	DLD	4	4.25	1.708	2–6
G: Questions/comments—things	T.D.	24	8.958	0.204	8–9
	DLD	4	6.75	2.062	4–9
H: Questions/comments—self/others	T.D.	24	34.208	2.604	27–36
	DLD	4	22.25	8.221	13–33
I: Talk in activities with others	T.D.	24	12.917	1.64	8–14
	DLD	4	12	3.367	7–14
J: Teasing/sense of humor	T.D.	24	4.167	1.049	2–5
	DLD	4	2.75	0.957	2–4
K: Interest in words and language	T.D.	24	10.5	1.445	8–12
	DLD	4	8.75	1.258	7–10
M: Adapting conversation to others	T.D.	24	12.833	2.353	7–15
	DLD	4	7.5	3.873	2–11
N: Building longer sentences and stories	T.D.	24	24	5.626	13–32
	DLD	4	10.25	8.655	2–20
		**Children aged 4 to 5 years**			
*N* total	T.D.	25			
	DLD	8			
MLUM	T.D.	25	5.267	1.352	2.108–7.2
	DLD	8	3.79	2.479	0–7.737
VOCD	T.D.	24	36.218	8.445	15.03–50.88
	DLD	8	25.181	17.859	0–42.75
Neighbourhood Game	T.D.	25	4.92	1.631	2–7
	DLD	8	3.625	2.446	0–6
Personal narrative	T.D.	25	4.96	1.274	2–7
	DLD	8	2.875	1.808	0–5
Language Use Inventory—French					
Total Score	T.D.	22	146.5	12.835	113–159
	DLD	8	113.25	23.438	85–148
Part 2: Word	T.D.	22	29.955	0.213	29–30
	DLD	8	27.75	2.915	22–30
Part 3: Longer sentences	T.D.	22	116.545	12.842	83–129
	DLD	8	85.5	22.367	55–119
C: Types of words	T.D.	22	23	0	23–23
	DLD	8	21	2.507	16–23
D: Requests for help	T.D.	22	6.955	0.213	6–7
	DLD	8	6.75	0.463	6–7
F: getting someone to notice something	T.D.	22	5.818	0.395	5–6
	DLD	8	5.375	0.518	5–6
G: Questions/comments—things	T.D.	21	8.905	0.301	8–9
	DLD	8	8.125	1.356	6–9
H: Questions/comments—self/others	T.D.	22	34.091	4.081	21–36
	DLD	8	27.875	8.061	14–36
I: Talk in activities with others	T.D.	22	13.182	1.332	9–14
	DLD	8	11.25	3.732	3–14
J: Teasing/sense of humor	T.D.	22	4.409	1.054	1–5
	DLD	8	2.75	1.035	2–5
K: Interest in words and language	T.D.	22	11.091	1.231	8–12
	DLD	8	7.25	2.252	4–11
M: Adapting conversation to others	T.D.	22	13.364	1.941	8–15
	DLD	8	8.875	3.182	3–13
N: Building longer sentences and stories	T.D.	22	26.091	5.309	12–32
	DLD	8	14	7.426	5–27

S.D. = standard deviation; T.D. = typically developing children; DLD = at risk for developmental language disorder group.

**Table 4:** Mean scores on each scale of the Social Competence and Behavior Evaluation Scale (raw scores).

Measure	Group	*N*	Mean	S.D.	Min.–Max.
		**Children aged 3 to 4 years**			
*N* total	T.D.	26			
	DLD	4			
General Adaptation	T.D.	25	313.96	33.35	202–361
	DLD	4	272.5	44.606	209–313
Social Competence	T.D.	25	143.76	17.484	103–175
	DLD	4	115.75	25.539	80–140
Internalizing Problems	T.D.	25	88.12	9.641	53–99
	DLD	4	74.75	19.585	46–90
Externalizing Problems	T.D.	25	82.08	11.496	46–95
	DLD	4	82	5.831	74–88
Depressive–Joyful	T.D.	25	42.04	3.857	33–47
	DLD	4	35.25	8.261	25–45
Anxious–Secure	T.D.	25	42.12	5.869	18–49
	DLD	4	36.25	9.43	23–45
Isolated–Integrated	T.D.	25	40.44	5.938	22–48
	DLD	4	33.5	11.03	18–44
Dependent–Autonomous	T.D.	25	38.52	4.51	26–46
	DLD	4	30.25	5.56	22–34
Aggressive–Calm	T.D.	25	40.76	3.503	30–47
	DLD	4	38.25	4.5	33–42
Egotistical–Prosocial	T.D.	25	33.76	6.47	20–44
	DLD	4	32.25	3.862	30–38
Angry–Tolerant	T.D.	25	40.76	3.503	30–47
	DLD	4	38.25	4.5	33–42
Oppositional–Cooperative	T.D.	25	40.8	5.523	23–47
	DLD	4	38.5	3.109	35–42
		**Children aged 4 to 5 years**			
*N* total	T.D.	25			
	DLD	8			
General Adaptation	T.D.	24	291.625	45.485	223–374
	DLD	8	279.75	60.937	156–362
Social Competence	T.D.	24	139.167	31.112	92–195
	DLD	8	133.5	42.173	41–182
Internalizing Problems	T.D.	24	82.5	8.617	59–94
	DLD	8	68.875	16.366	43–90
Externalizing Problems	T.D.	24	69.958	15.741	36–96
	DLD	8	77.375	8.141	71–90
Depressive–Joyful	T.D.	24	38.958	6.245	24–46
	DLD	8	35.125	9.265	17–49
Anxious–Secure	T.D.	24	41.708	7.025	20–49
	DLD	8	36.125	9.296	21–49
Isolated–Integrated	T.D.	24	39.542	6.966	23–48
	DLD	8	33	9.381	11–42
Dependent–Autonomous	T.D.	24	38.292	6.798	22–48
	DLD	8	29.75	11.925	5–43
Aggressive–Calm	T.D.	24	35.917	7.667	18–48
	DLD	8	40.5	3.586	33–44
Egotistical–Prosocial	T.D.	24	29.583	8.89	14–44
	DLD	8	34.75	7.206	26–47
Angry–Tolerant	T.D.	24	29.708	10.084	15–49
	DLD	8	30.875	10.467	12–47
Oppositional–Cooperative	T.D.	24	37.917	7.168	25–50
	DLD	8	39.625	8.585	24–49

S.D. = standard deviation; T.D. = typically developing children; DLD = at risk for developmental language disorder group.

**Table 5:** BF_10_ values for t-tests comparing the results between the typical language and at risk for DLD groups on all language and social competence measures.

Task	Measure	BF_10_	Interpretation—Strength of Evidence
Linguistic measures from personal narrative	MLUM	2.837	Anecdotal in favor of H_1_ ^1^
	VOCD-D	3.695	Moderate in favor of H_1_
Neighbourhood Game	Communicative intents	6.232	Moderate in favor of H_1_
Language Use Inventory– French	Total score	23.992	Strong in favor of H_1_
	Part 2: Words	5.339	Moderate in favor of H_1_
	Part 3: Longer sentences	76.731	Strong in favor of H_1_
	C: Types of words	12.351	Strong in favor of H_1_
	D: Requests for help	0.637	Anecdotal in favor of H_0_
	F: Getting someone to notice something	2.486	Anecdotal in favor of H_1_
	G: Questions/comments—things	1.033	Anecdotal in favor of H_1_
	H: Questions/comments—self/others	11.013	Strong in favor of H_1_
	I: Talk in activities with others	1.19	Anecdotal in favor of H_1_
	J: Teasing/sense of humor	14.426	Strong in favor of H_1_
	K: Interest in words and language	15.846	Strong in favor of H_1_
	M: Adapting conversation to others	32.186	Strong in favor of H_1_
	N: Building longer sentences and stories	37.675	Strong in favor of H_1_
Personal narrative	High point	11.576	Strong in favor of H_1_
Social competence and behavior evaluation	General Adaptation	0.684	Anecdotal in favor of H_0_
	Social Competence	0.641	Anecdotal in favor of H_0_
	Internalizing Problems	4.019	Moderate in favor of H_1_
	Externalizing Problems	0.192	Moderate in favor of H_0_
	Depressive–Joyful	0.992	Anecdotal in favor of H_0_
	Anxious–Secure	1.287	Anecdotal in favor of H_1_
	Isolated–Integrated	1.565	Anecdotal in favor of H_1_
	Dependent–Autonomous	10.739	Strong in favor of H_1_
	Aggressive–Calm	0.234	Moderate in favor of H_0_
	Egotistical–Prosocial	0.169	Moderate in favor of H_0_
	Angry–Tolerant	0.417	Anecdotal in favor of H_0_
	Oppositional–Cooperative	0.288	Moderate in favor of H_0_

^1^ H_1_ indicates that the typical language group obtained higher scores than the at risk for DLD group. * Missing data for the typical language group: 1 for MLUM, Neighbourhood Game, personal narrative, and SCBE; 2 for VOCD-D, 5 for LUI-French. MLUM = mean length of utterances in morphemes.

**Table 6:** Correlations between the Social Competence and Behavior Evaluation (SCBE) scales and language measures for which the strength of evidence is at least moderate (BF_10_ ≥ 3, n = 63).

Social Competence and Behavior	Language Measures								
	MLUM	VOCD-D	LUI-French Subscale I	LUI-French Subscale J	LUI-French Subscale K	LUI-French Subscale M	LUI-French Part 3	LUI-French Total Score	Personal Narrative
**General** **Adaptation**						τ*b* = 0.24 * BF_10_ = 5.565			
**Social** **Competence**						τ*b* = 0.234 * BF_10_ = 4.606			
**Internalizing** **Problems**				τ*b* = 0.238 * BF_10_ = 5.228	τ*b* = 0.34 ** BF_10_ = 178.78	τ*b* = 0.317 ** BF_10_ = 71.858	τ*b* = 0.246 * BF_10_ = 6.515	τ*b* = 0.239 * BF_10_ = 5.384	τ*b* = 0.22 * BF_10_ = 3.434
**Externalizing** **Problems**			τ*b* = −0.255 * BF_10_ = 8.495						
**Depressive–Joyful**						τ*b* = 0.229 * BF_10_ = 3.997			τ*b* = 0.229 * BF_10_ = 4.427
**Anxious–Secure**					τ*b* = 0.247 * BF_10_ = 6.672	τ*b* = 0.218 * BF_10_ = 3.271	τ*b* = 0.24 * BF_10_ = 5.494	τ*b* = 0.235 * BF_10_ = 4.797	
**Isolated–Integrated**					τ*b* = 0.233 * BF_10_ = 4.463	τ*b* = 0.291 ** BF_10_ = 18.509	τ*b* = 0.242 * BF_10_ = 5.828	τ*b* = 0.234 * BF_10_ = 4.661	τ*b* = 0.234 * BF_10_ = 5.214
**Dependent–Autonomous**	τ*b* = 0.24 * BF_10_ = 6.174	τ*b* = 0.283 ** BF_10_ = 22.806		τ*b* = 0.281 ** BF_10_ = 20.11	τ*b* = 0.289 ** BF_10_ = 25.853	τ*b* = 0.377 ** BF_10_ = 887.427	τ*b* = 0.286 ** BF_10_ = 23.898	τ*b* = 0.274 ** BF_10_ = 15.916	τ*b* = 0.276 ** BF_10_ = 19.496
**Oppositional–Cooperative**						τ*b* = 0.22 * BF_10_ = 3.131			

*Note*: * = weak correlation; ** = moderate correlation; *** = strong correlation; τ*b* = Kendall’s tau-b.

## Data Availability

The data presented in this study are available on request from the corresponding author due to ethical restrictions.

## Note

1 In Quebec, children under 5 years old who have significant language difficulties usually receive an “at risk of DLD” diagnosis. The diagnosis of DLD is confirmed or invalidated according to the child’s progress following speech therapy.

## Abbreviations

The following abbreviations are used in this manuscript:

BF	Bayes factor
DLD	Developmental language disorder
ICF	International Classification of Functioning, Disability and Health
LUI-French	Language Use Inventory: French
MLUM	Mean length of utterances in morphemes
PN	Personal narrative
SCBE	Social Competence and Behavior Evaluation Scale
